# Clinical relevance of contextual factors as triggers of placebo and nocebo effects in musculoskeletal pain

**DOI:** 10.1186/s12891-018-1943-8

**Published:** 2018-01-22

**Authors:** Giacomo Rossettini, Elisa Carlino, Marco Testa

**Affiliations:** 1 Department of Neuroscience, Rehabilitation, Ophthalmology, Genetics, Maternal and Child Health, University of Genova, Campus of Savona. Via Magliotto, 2, 17100 Savona, Italy; 2 Department of Neuroscience, University of Turin Medical School, Turin, Italy

**Keywords:** Placebo, Nocebo, Contextual factors, Clinical reasoning, Pain, Expectation, Conditioning, Learning, Therapeutic encounter, Therapeutic relationship

## Abstract

Placebo and nocebo effects are embodied psycho-neurobiological responses capable of modulating pain and producing changes at different neurobiological, body at perceptual and cognitive levels. These modifications are triggered by different contextual factors (CFs) presented in the therapeutic encounter between patient and healthcare providers, such as healing rituals and signs. The CFs directly impact on the quality of the therapeutic outcome: a positive context, that is a context characterized by the presence of positive CFs, can reduce pain by producing placebo effects, while a negative context, characterized by the presence of negative CFs, can aggravate pain by creating nocebo effects. Despite the increasing interest about this topic; the detailed study of CFs as triggers of placebo and nocebo effects is still lacked in the management of musculoskeletal pain.

Increasing evidence suggest a relevant role of CFs in musculoskeletal pain management. CFs are a complex sets of internal, external or relational elements encompassing: patient’s expectation, history, baseline characteristics; clinician’s behavior, belief, verbal suggestions and therapeutic touch; positive therapeutic encounter, patient-centered approach and social learning; overt therapy, posology of intervention, modality of treatment administration; marketing features of treatment and health care setting. Different explanatory models such as classical conditioning and expectancy can explain how CFs trigger placebo and nocebo effects. CFs act through specific neural networks and neurotransmitters that were described as mediators of placebo and nocebo effects.

Available findings suggest a relevant clinical role and impact of CFs. They should be integrated in the clinical reasoning to increase the number of treatment solutions, boosts their efficacy and improve the quality of the decision-making. From a clinical perspective, the mindful manipulation of CFs represents a useful opportunity to enrich a well-established therapy in therapeutic setting within the ethical border. From a translational perspective, there is a strong need of research studies on CFs close to routine and real-world clinical practice in order to underline the uncertainty of therapy action and help clinicians to implement knowledge in daily practice.

## Background

Pain represents a “distressing experience associated with actual or potential tissue damage with sensory, emotional, cognitive and social components” [1]. Among the different pain conditions, musculoskeletal pain is ubiquitous and multifaceted: it can be the consequence of everyday activities that repeatedly or unusually stress the system, or it can be due to either acute traumatic events or to musculoskeletal diseases [2]. It is the most disabling symptom in musculoskeletal disorders, causing a high number of requests for healthcare treatments and rising social costs [3]. Moreover, especially in chronic conditions when pain persists beyond the normal healing time, it is influenced by different physical, psychological and social factors [4–6] defined as “contextual factors” (CFs).

The multidimensionality that characterizes pain in musculoskeletal complaints requires an integrative and personalized approach for its treatment. For this reason, the study of the CFs and their conscious use and integration in the clinical practice could represent a novel approach in the management of this complex experience [7–16].

By definition, CFs are physical, psychological and social elements that characterize the therapeutic encounter with the patient [17, 18]. CFs are actively interpreted by the patient and are capable of eliciting expectations, memories and emotions that in turn can influence the health-related outcome, producing placebo or nocebo effects [19]. In other words, the CFs represent the context that accompany any healthcare treatment: the exposure of a patient to a positive context (positive CFs) very often produces a placebo effect that is the occurrence of symptoms improvement (e.g. analgesia), whereas a negative context (negative CFs) can generate a nocebo effect, with a worsening of the pain condition (e.g. hyperalgesia) [20, 21]. In the following review, we use the term CFs instead of placebo, avoiding the misleading interpretation of placebo as inert treatment given to comfort or please the patient and following the recent conceptualization of the placebo as the psychosocial context that accompanies any medical intervention, be it active or sham [22–31].

As extensively demonstrated by the placebo and nocebo effect literature, the CFs can affect the outcome of a treatment with different mechanisms and in different systems, medical conditions, and therapeutic interventions [32]. From a clinical perspective, the study of CFs as triggers of placebo and nocebo effects, is crucial for the management of musculoskeletal pain for several reasons [33]. First, even if CFs are embodied in every complex therapeutic interventions in musculoskeletal complaints, they are often considered as incidental factors capable to affect outcomes. For this reason they are not always identified and used intentionally by clinicians [34]. Second, CFs can produce a therapeutic effect through the involvement of the same central pathways of pain modulation activated by several hands-on (e.g. manual therapy, therapeutic exercises, acupuncture, injections) and hands-off solutions (e.g. pain neuroscience education) commonly applied in clinical practice [35–37]. Third, CFs serve as additional tools for the interpretation of the clinical picture and guide clinicians in managing the complexity behind the patient’s musculoskeletal pain [38]. Taking into consideration CFs as active influencer of the therapeutic outcomes, can help to explain some unexpected outcomes and variability of symptoms experience [39].

Moving from this vision, the present debate is proposed to all the health professionals (physiotherapists, chiropractors, osteopaths, nurses, occupational therapists, rheumatologists, orthopedics etc.) that work with musculoskeletal pain. In order to support a better and more conscientious therapeutic use of the CFs in musculoskeletal field, the purposes of this debate are to: 1) briefly define the CFs, how they work and act from a neurophysiological perspective; 2) underline their clinical relevance in pain management; 3) consider their role in clinical reasoning, within the ethical border and 4) suggest how to take them into account in the research field.

## Contextual factors

### What do the contextual factors represent?

A treatment is never administered in a neutral situation, but rather in a complex set of CFs, that Balint called the “atmosphere around the treatment” [40] and Miller and Kaptchuk called “contextual healing” [41]. Following these definitions, it is clear that the CFs can act “independently” by the nature of the treatment: since they represent the context of any medical treatment, they have a role when a sham treatment is administered but also when an active treatment is administered.

CFs were introduced in 2001 by Di Blasi et al. [17] in medical community and recently exploited by Testa & Rossettini in physiotherapy field [33]. CFs can be internal, external or relational. The internal factors consist of memories, emotions, expectations and psychological characteristics of the patient; the external factors include the physical aspects of therapy, such as the kind of treatment (pharmacological or manual) and the place in which the treatment is delivered. Relational factors are represented by all the social cues that characterizes the patient-physiotherapist relationship, such as the verbal information that the physiotherapist gives to the patient, the communication style or the body language [19].

A clear identification of the CFs is crucial in clinical practice, in order to enhance the treatment efficacy. In a work targeted to physiotherapy field, CFs have been grouped in 5 different categories on the base of their sensory and social features [33]: physiotherapist characteristics (professional reputation, appearance, beliefs, behaviours); patient characteristics (expectation, preferences, previous experience, musculoskeletal condition, gender, age); patient-physiotherapist relationship (verbal communication, non-verbal communication), treatment (clear diagnosis, overt therapy, observational learning, patient-centered approach, global process of care, therapeutic touch), healthcare setting (environment, architecture, interior design).

During any clinical phase (e.g. consultation, examination and treatment) the CFs “inform” the patient that a healthcare procedure has been delivered and they could positively or negatively affect symptom perception, experience and meaning [20, 21].

The identification of the CFs and the attention to healthcare context is crucial for at least two reasons. First, a treatment delivered in a positive context (positive CFs) produces better outcomes than a treatment delivered in a neutral condition or negative context (negative CFs). The open-hidden approach is one of the best evidence of decreased effectiveness of a medical treatment when a meaningful context is eliminated [42]. In the “open” condition, that mimics the routine medical practice, a treatment is delivered in full view of the patient: it means that the patient is aware of receiving a medical treatment and know when the medical treatment is delivered. In the “hidden” condition, the treatment is administered unbeknownst to the patient. Different studies have reported that open treatments are more effective than hidden treatments, because in the hidden condition the surrounding context (healing rituals, therapist-patient interaction, etc.) is absent, thus losing its positive meaning [43–46].

Second, the psychosocial context can influence the patients in different ways since the responsiveness to the context seems to be not a stable trait but a situational trait [47], and the same patient can sometimes positively respond to the context and sometimes not. Thus, if a patient is not influenced by the therapeutic context (the so called “placebo non-responders”) he/she needs more medical attention because the lower the placebo responsiveness, the lower the treatment responsiveness [48]. Indeed, if the total treatment effect is conceptualized as the sum of the CFs effect plus the active treatment effect plus the interaction of the CFs and active treatment effects [49], a patient that is not sensible to the positive influence of the CFs will show a lower treatment response [50].

### How do the contextual factors trigger placebo and nocebo effects?

If we aim to implement an aware use of CFs along the clinical routine, the understanding of how they work has a capital importance. The CFs shape placebo and nocebo effects through different sources. Historically, the most important models include classical conditioning and expectation processes.

Following the *classical conditioning*, different external CFs represent an example of conditioned stimuli that evoke a conditioned response [51]. In general, as proposed by this model, the repeated contingency between a salient unconditioned stimulus (e.g., sight of food) with a neutral conditioned stimulus (e.g., a bell ringing) can induce the same conditioned response (i.e., salivation) even if the neutral stimulus is presented alone. In the specific contest of healthcare, different aspects of the healthcare setting or physical features of the medical treatment can act as external conditioned stimuli, eliciting a therapeutic response in the absence of an active principle, just because they have been previously associated with it. Recently, other learning mechanisms has been documented, such social learning. In particular, beyond direct first-hand experience to specific external CFs, it is possible to learn a conditioned response by observing other people that respond to specific CFs [9].

Following the *expectation model*, different external, internal and relational CFs can activate the expectancy of pain relief, triggering neurobiological changes and symptoms’ amelioration [52]. Verbal suggestions are typical external CFs that trigger positive or negative responses. For example, the administration of an analgesic treatment along with the expectations of pain relief can lead to a positive analgesic response, whereas the administration of an analgesic treatment without specific expectations or with expectations or pain exacerbation can result in a negative response and in the perpetration of pain [53].

**Fig. 1:**
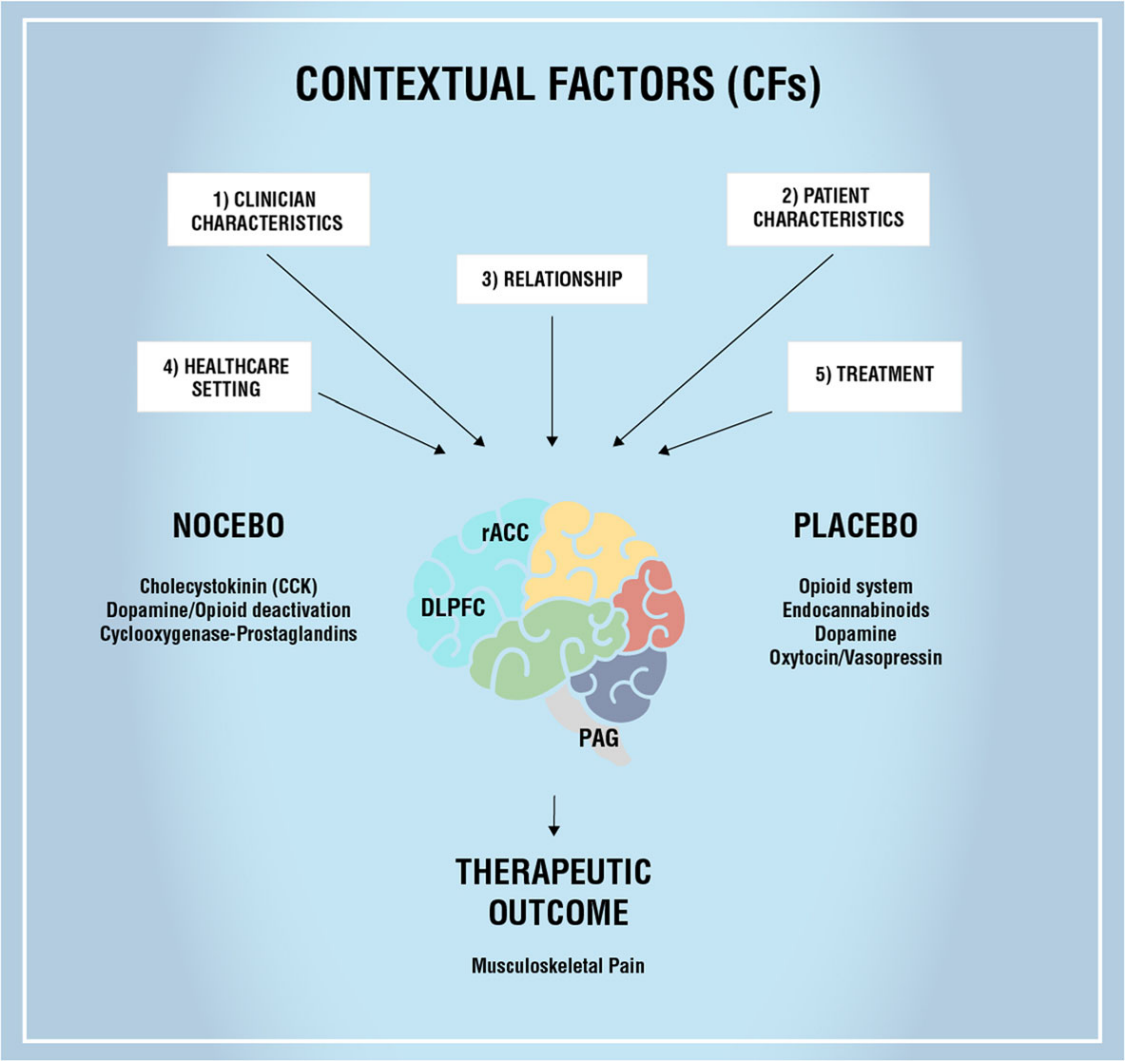
Psycho-neurobiological mechanism of CFs. The image displays how CFs are capable to influence the brain networks, neurochemistry and therapeutic outcome. The principal neural areas and neurotransmitters involved in placebo and nocebo effects are reported. Abbreviation: rACC = Rostral Anterior Cingulate Cortex; DLPFC = Dorsolateral prefrontal cortex; PAG = Periaqueductal gray

Following the Colloca and Miller integrative model [54], conditioning and expectations are not mutually exclusive and can be integrated in a more general learning model, whereby various types of CFs trigger expectancies, memories and emotions that in turn generate behavioral and clinical outcome changes, through the activation of the central nervous system (Fig. 1) [7, 9, 20, 21]. In other words, the presence of external CFs, combined with specific internal and relational CFs, is interpreted by the patient and converted into neural input events and behavioral changes [54]. This model represents a good conceptualization of the role of the therapeutic context, useful also at the clinical practice level. Indeed, it opens up to the possibility to study the effects and the impacts of every single CF on the outcome of a medical treatment.

### How do the contextual factors work at the neurobiological level?

A robust body of knowledge, especially acquired in the field of pain, has identified the neural networks activated by the CFs. Indeed, a crucial question that catch the attention of neuroscientists and clinicians is whether the subjective changes in the outcome after the exposure to a specific therapeutic context are associated with specific neurobiological activities [10]. Pharmacological studies, as well as neuroimaging studies, have address this question using different experimental approaches based on classical conditioning and modulation of expectations. Taken together, these studies demonstrated that different changes in the pain processing network occurs when positive or negative CFs trigger placebo or nocebo effects, respectively. In particular, pain reduction is associated with decreased activity in the classical pain-matrix areas, such as the thalamus, insula, somatosensory cortex, and mid-cingulate regions [55–60]. Interestingly, positron emission tomography (PET) studies showed that the analgesic effect induced by the administration of a real mu-agonist, such as remifentanil, and the analgesic effect triggered by verbal suggestions determined similar activation of different brain regions, such as rostral anterior cingulate cortex and the orbital cortex [61, 62]. Separating the pain anticipation phase and the pain perception phase, a meta-analysis of brain imaging data using the activation likelihood estimation method, identified the involvement of different brain regions: during expectation, areas of activation are found in the anterior cingulate, precentral and lateral prefrontal cortex, and in the periaqueductal gray, whereas during pain inhibition, deactivations are found in the mid- and posterior cingulate cortex, superior temporal and precentral gyri, in the anterior and posterior insula, in the claustrum and putamen, and in the thalamus and caudate body [63]. On the other hand, pain increase is associated with signal increases in several regions including anterior cingulate cortex, insula, left frontal and parietal operculum [64–67]. Also, high temporal resolution techniques, such as electroencephalography (EEG), have confirmed that the amplitude of specific evoked potentials, both related to pain anticipation and to pain perception, are affected by the CFs [68–71]. Thus, both early and late sensory components of pain processing are affected by the exposure to positive and negative CFs.

Different studies have also characterized the neurotransmitter systems activated the CFs. Using a classical conditioning approach, it has been demonstrated that when an opioid drug, such as morphine, is delivered for different days and then it is replaced by a placebo unbeknownst to the patient a placebo analgesic effect occurs [72]. This effect can be blocked by the mu opioid antagonist, naloxone, thus indicating that the opioid system plays an important role [57, 73, 74]. An indirect evidence of the involvement of the opioid system comes from the study of the anti-opioid action of the cholecystokinin (CCK) system. The proglumide, that is a CCK antagonist, enhances placebo analgesia [75, 76], whereas the activation of the CCK type-2 receptors with the agonist pentagastrin disrupts it [77]. These pharmacological data have been confirmed by a neuroimaging study, in which the authors proved that naloxone blocked the placebo analgesic response in dorsolateral prefrontal cortex (DLPFC), rostral anterior cingulate cortex (rACC), hypothalamus, periaqueductal gray (PAG), and rostral ventromedial medulla (RVM), and abolished placebo-enhanced coupling between rACC and PAG [57]. Using a the same conditioning protocol, it has been demonstrated that also the cannabinoid system is activated by the positive therapeutic context: when non-opioid drugs, like ketorolac, are administered for 2 days in a row and then replaced with a placebo on the third day, the analgesic effect is not reversed by naloxone, whereas the CB1 cannabinoid receptor antagonist, rimonabant, blocks this placebo analgesia completely [78]. Also studies in which expectations were manipulated by positive verbal suggestions, showed an activation of μ-opioid neurotransmission in the dorsolateral prefrontal cortex, the anterior cingulate cortex, the insula, and the nucleus accumbens [79, 80].

A different system activated by the therapeutic context is the dopaminergic system: indeed, the positive effect due to the presence of positive CFs seems to be related to the activation of dopamine in the nucleus accumbens. as assessed using in vivo receptor binding PET with raclopride. Moreover, when expectations of pain reduction were induced, the analgesic effect of the context was associated with activation of opioid neurotransmission in the anterior cingulate, orbitofrontal and insular cortices, nucleus accumbens, amygdala, and periaqueductal gray matter. Dopaminergic activation was observed in the ventral basal ganglia, including the nucleus accumbens. Both dopaminergic and opioid activity were associated with both anticipation and perceived effectiveness of the positive verbal suggestions [81, 82].

Recently, oxytocin [83] and vasopressin [84] have been identified CFs enhancer as they potentiate the analgesic effect due to the presence of positive verbal suggestions. Moreover it has been documented that negative expectations about headache pain led to the enhancement of the cyclooxygenase-prostaglandins pathway, which, in turn, induced pain worsening [85].

## Clinical relevance of the contextual factors

### What is the magnitude of placebo and nocebo effects induced by CFs in musculoskeletal pain?

The impact of CFs as trigger of placebo and nocebo effects on pain outcome has been quantified in different ways and has been reported in a wide range of musculoskeletal conditions such as low back pain [86–108], neck pain [95, 99, 109–111], shoulder pain [95, 112, 113], osteoarthritis [38, 91, 99, 100, 114–125], rheumatoid arthritis [126], and fibromyalgia [97, 127–132].

Different studies have measured the magnitude of placebo and nocebo effects induced by CFs in different musculoskeletal pain conditions commonly encounter in daily setting [117, 133]. Indeed the clinical effectiveness of placebo analgesia was demonstrated in specific complaints such as fibromyalgia [128] and osteoarthritis [118] with an effect size (ES) over 0.5. Also, nocebo hyperalgesia measured as dropout rate due to adverse event were present in fibromyalgia (9.6%) [134] and osteoarthritis (4.8%) [135]. Concerning osteoarthritis, the ES decreased consistently from hand, to knee, to combined hip and knee and then to hip [118, 136].

Moreover, considering the overall treatment efficacy as the sum of the specific component related to the active treatment plus the unspecific component due to the CFs, the impact of the CFs was measured in different conditions and interventions [137]. Zou and colleagues showed that 75% of the overall treatment effect in osteoarthritis is attributable to contextual effects rather than the specific effect of treatments [116]. In fibromyalgia, the 45% of the response of the active drug is attributable to contextual effect [129] and a relevant contextual effects was shown also in aspecific low back pain [138]. Moreover, a recent meta-analysis on spinal manual therapies showed that in acute pain and chronic pain, respectively 81 and 66% of the pain variance were ascribed to CFs [139].

### Which kind of CFs influence musculoskeletal pain conditions?

Considering the patient’s perspective, *expectations toward the therapy, patient’s treatment history and baseline pain severity* are elements capable to predict the outcomes of different musculoskeletal pain treatments.

Expectations of symptoms improvement can be activated by different CFs: for example, the simple act of administering a treatment, the exposure to a clinical setting, the verbal or non-verbal interaction with the physician are capable of triggering patient’s expectations. As demonstrated by different studies, boosting patient’s expectations toward the therapy significantly increased the chance of pain relief more than delivering a treatment without the expectation of any benefit [86, 90–98, 109, 111, 112, 126, 140].

Patient’s treatment history, that is the patient’s history of past positive or negative medical treatments, can influence the future response of the patient to new medical treatments. Previous positive experiences obtained by a specific therapy increase the likelihood of future positive experiences with the same therapy, while precedent negative outcomes associated to a particular intervention increase the probability of negative outcomes [99, 141].

Higher pain intensity at baseline [99, 100, 118, 128, 129] and the presence of concomitant diseases and psychosocial elements such as depressions [99, 130] are associated with an augmented placebo analgesia and reduced nocebo hyperalgesia. Long-term dysfunction seems to respond less to placebo analgesia indicating that duration of complaints influence placebo analgesia [128, 132].

From the provider’s perspective, *clinician’s behavior, belief, verbal suggestions and therapeutic touch* can strongly influence patients’ pain perception.

A provider acting as competent, experienced, educated, professional, trustworthy, capable to indicate a diagnosis and prognosis, and to monitor patient with follow up, can moderate pain with his behavior [38, 114, 115, 120, 142]. Aligning his/her beliefs with patient’s beliefs, a clinician could modulate pain. Indeed, it was demonstrated that the healthcare provider’s point of view concerning the clinical pathway, the therapy and the prognosis influence patient’s pain [38, 101–103, 107, 114, 115, 143, 144]. Informing the patient that a potent treatment has been delivered enhanced the analgesic effect of the treatment, conversely verbal suggestion concerning the threatening effect of the therapy can compromise the effectiveness of the treatment creating nocebo hyperalgesic effects [108, 121, 122, 141, 145, 146]. Non-verbal communication has powerful effects as well. For example, the use of therapeutic touch can positively influence patient’s pain [105, 123, 124, 131, 147, 148].

Finally, considering the patient-physician relationship, it appears that a *positive therapeutic encounter* between patient and clinicians can lead to additional clinical benefits. Indeed, an enhanced empathetic interaction comprehensive of therapeutic alliance, active listening, extra time spent with patient, more face-to-face visit, warmth, attention, care, encouragement and support significantly reduced pain more than the same therapy performed with neutral therapeutic interaction [87, 88, 100, 104, 145, 149, 150]. Moreover, a *patient-centered approach* can increase the effectiveness of the therapy. Indeed, the patient’s involvement in the global process of care has been shown to modulate pain [106, 125]. The strategy to favor the *social learning* between patients by the observation of other’s pain improvement or reduction is capable to affect the observers’ symptomatology [151, 152].

Also the way by which the therapy is administered can influence pain perception. The adoption of an *overt paradigm* that enhances patient’s knowledge of being treated modulates the therapeutic outcome [110]: a significant pain reduction was observed after the execution of an exercise in an environment that allowed patients to visualize their body [89]. Also the *posology of intervention* has an effect as CFs: the placebo effect is higher when therapies are more frequent and repeated a therapy is delivered (e.g. two or more times vs one time) [118]. The choice of the *modality of treatment administration* can be crucial to modulate patient’s pain. In general, the higher is the invasiveness of treatment (e.g. acupuncture, dry needling, injection, surgery), the better is the reduction of pain [116, 118, 119, 153, 154]. Moreover, parenteral or subcutaneous administrations (e.g. topical) are more efficient than oral administrations [115, 116, 119].

Even the *marketing features of treatment* should be taken into account. Branded therapy seems to be more effective than unbranded therapy [114, 115]. High prize medication produced better pain relief then discounted medication, therapy considered as “new” improved pain more than “usual” therapy [114, 115]. The more complex is the procedure including therapeutic rituals, mysterious powers, high technology the larger the placebo effect [114, 115].

Lastly, the *health care setting*, in terms of environment, architecture and interior design should not be overlooked. The use of facilities where evidence-based design such as furnishing, colors, artwork, light, outside views, temperature, soothing sound and music were adopted, positively impacts on patient’s pain creating a proper healing setting [127, 155–157].

## Clinical applications and translational research

### Is it time to implement CFs in our clinical reasoning?

The clinical reasoning adopted by clinicians in musculoskeletal conditions represents a complex procedure that encompasses different dimensions of pain experience in a bio-psycho-social framework [158]. Indeed, this multi-factorials thinking process considered biomedical (e.g. tissue pathology, disease), psychological and social elements (e.g. experience of disability, patient’s belief, values and perspective) to obtain more complete analyses of the patient’s dysfunction [159]. The role and the impact of CFs should be integrated in the clinical reasoning to increase the number of treatment solutions, boosts their efficacy and improve the quality of the decision-making [33]. Based on the evidence available, some considerations can be drawn to guide a more conscious use of CFs as activators of placebo analgesia and avoiders of nocebo hyperalgesia.

Considering the *global process of care*, clinicians should be aware that the overall therapeutic outcome is determined by the suitability of the therapy adopted (“what we do”) and by how it is delivered (“how we do”) [33]. In this perspective, every musculoskeletal pain treatment is composed by a specific component and by a contextual component [34]. These components represent the two faces of the same coin and are capable of influencing pain at multiple levels of the central nervous system [160]. The use of the best evidence-based therapy is unquestionable, but clinicians should not forget the role of the CFs, as the context surrounding the specific treatment is capable of generating placebo or nocebo responses and modifying the therapeutic trajectory towards a positive or a negative direction [42].

Because it is a fact that placebo [161–163] and nocebo [164] effects are always present in routine clinical practice and can be triggered by CFs [33], clinicians should be able to use them to optimize the results and reduce failures. Indeed, there are clear evidence that, when placebo was purposely searched as a mechanism, the effect size was about five times greater (Cohen’s d ranging from 0.95 to 1.14) [161–163] than when placebo was used as a control condition (Cohen’s d ranging from 0.15 to 0.27) [165–167]. Moreover, clinicians should combine at the same time different CFs to obtain a larger placebo effect and minimize the nocebo effects. Some studies demonstrated that a lower effect size is present when using verbal suggestions alone (placebo - Cohen’s d = 0.85; nocebo - Cohen’s d = 0.65), while a higher effect size was observed adopting a combination of verbal suggestions and conditioning procedures (placebo - Cohen’s d = 1.45; nocebo - Cohen’s d = 1.07) [161, 164].

**Fig. 2:**
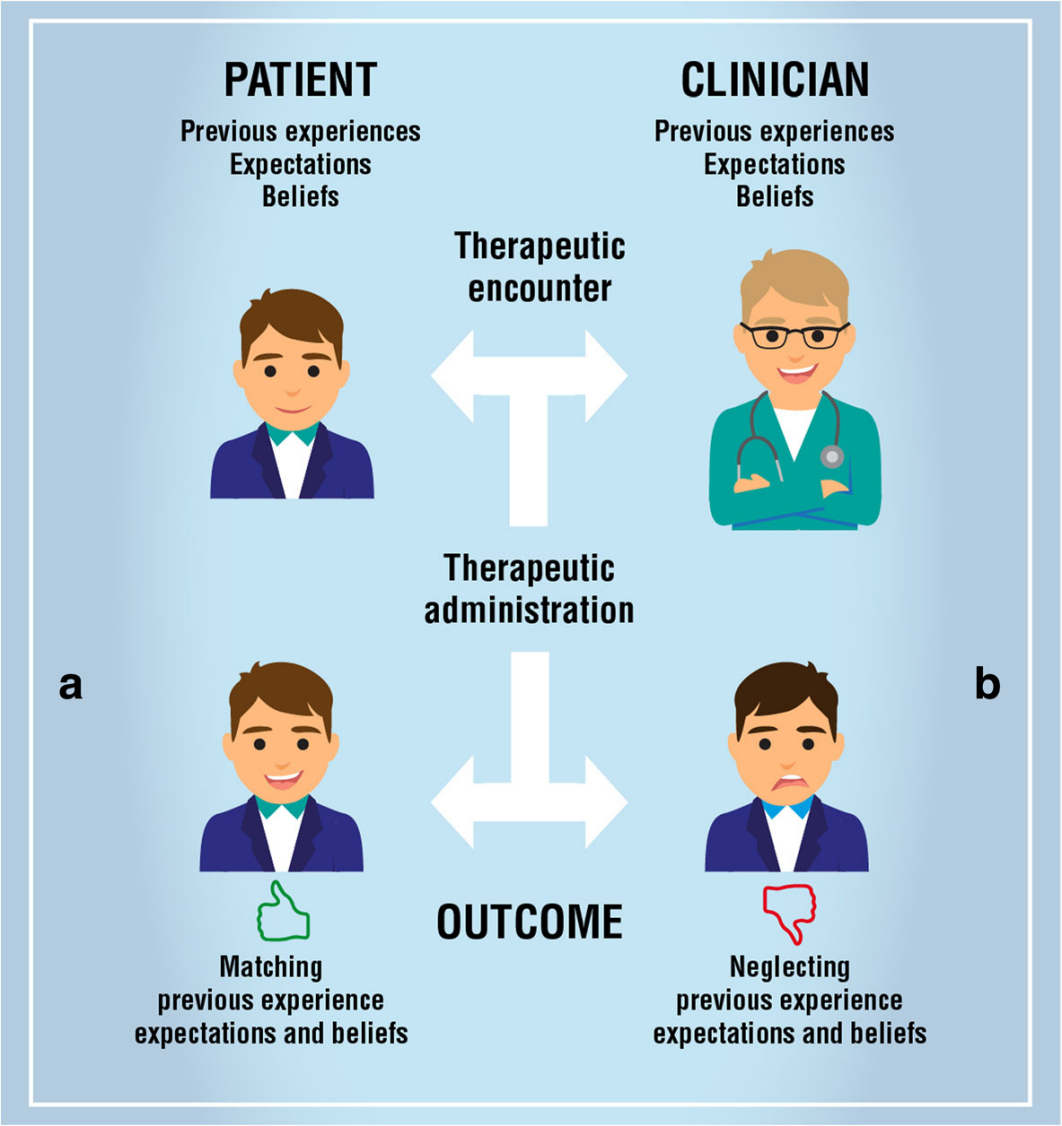
Influencers of decision-making process. The image presents: **a** the clinical situation in which meeting patient’s expectation, previous experience and beliefs creates positive therapeutic outcomes; **b** the clinical situation in which ignoring patient’s expectation, previous experience and beliefs creates negative therapeutic outcomes

Since placebo effects are learning phenomenon [9], during the *history taking*, clinicians should assess the patient’s previous experience, expectations and beliefs giving the patient adequate time to tell his/her story [50, 168, 169] (Fig. 2).

Previous successful and unsuccessful experiences of a specific treatment are capable to influence the therapeutic outcome [170]. In order to plan a therapeutic intervention, it’s important to question about past memories of analgesic and hyperalgesic responses concerning a treatment; reinforcing the positive experiences and devaluating the negative ones [7, 169, 171–173]. For example, if a patient had a previous negative experience with a specific treatment, clinician should avoid adopting it. On the contrary, if a patient experienced a positive outcome with a treatment, the use of the very same treatment is recommended in order to “activate” the patient’s positive memory of the previous treatment.

Since patients’ expectations about the therapeutic benefit influence the effectiveness of the treatment, a clear assessment of patients’ expectations toward the therapy is crucial. In particular, it is crucial to identify patients with low expectations in order to work with them with the aim of improving their belief [174]. Different scale and semi-standardized questionnaires have been proposed to assess patient’s expectations. For example, Younger et al. developed a tool for measuring patient outcome expectancy. The authors found that the final six-item scales, made of two subscales (positive expectancy and negative expectancy), predicted a significant amount of outcome variance in patients receiving surgical and pain intervention [175].

Moreover, clinicians should monitor patient’s belief concerning musculoskeletal conditions, therapeutic action, prognosis and ask questions about the meaning they attribute to symptoms [169, 172, 176–178]. In these times of important expansion of healthcare information delivering by Internet, social media and television it is crucial to avoid the misinformation [7]. The discussion with the patients can help the clinician to guide them to evidence-based information and avoid that they refer to unproven or fake information [176]. Also, asking systematically the patients to summarize the information provided can prevent negative misunderstandings about their complaints [169, 173, 176, 178].

In the *waiting, examination, therapeutic and follow-up phases*, the social interaction between patients [179, 180], the therapeutic ritual [181, 182] and the awareness of the ongoing procedure [42, 43] are fundamental elements to consider.

**Fig. 3:**
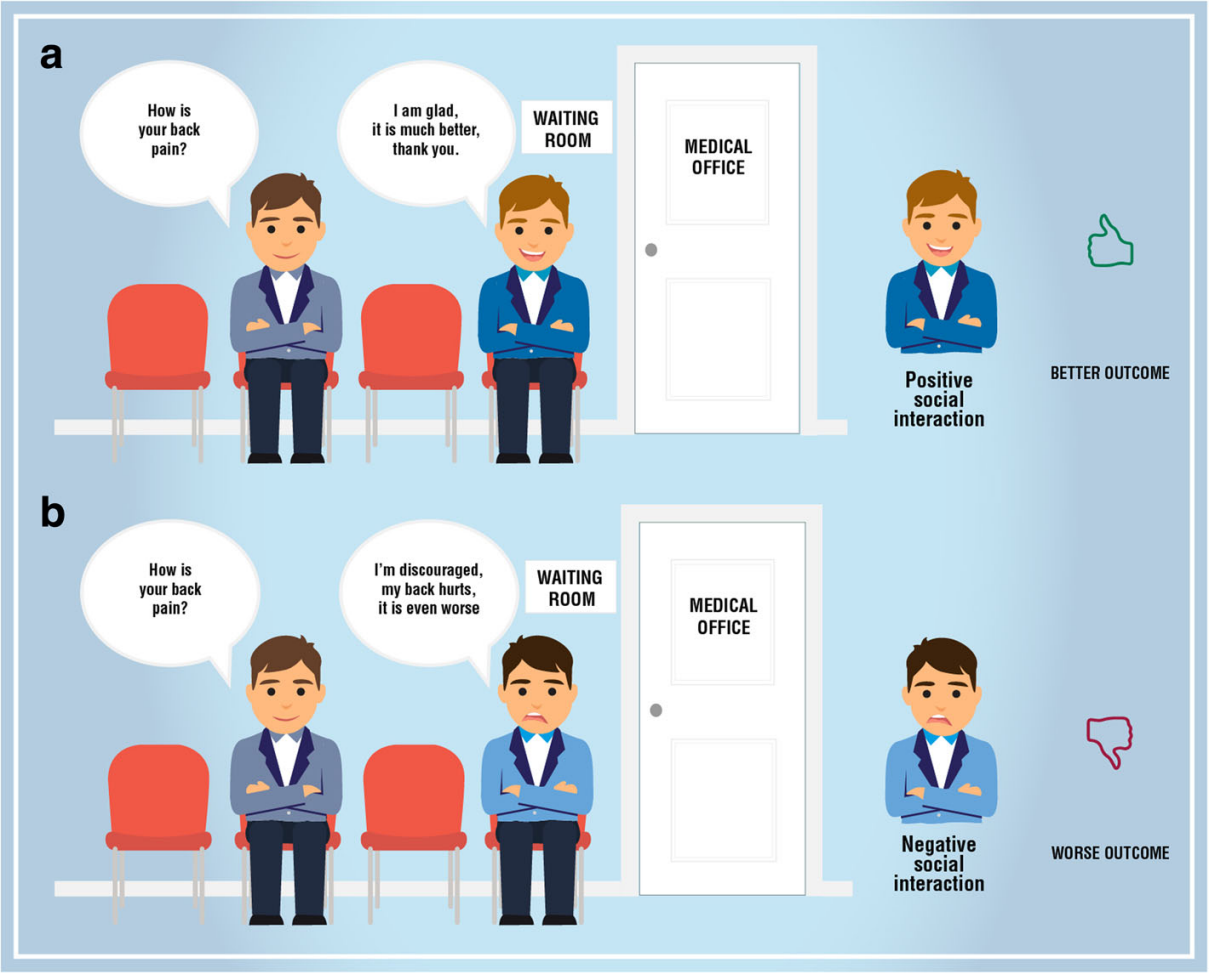
Social interaction and learning. The image displays: **a** a positive social interaction between patients in waiting room capable to produce positive therapeutic outcome; **b** a negative social interaction between patients in waiting room capable to produce negative therapeutic outcome

While waiting for healthcare encounter, a pleasant and peaceful environment, employing professional, friendly and helpful support staff can help patients to feel comfortable [169]. In waiting rooms, clinicians should reduce the social contagion of negative emotions preventing the patient’s interaction and/or observation of another patient experiencing a negative outcome (e.g. increased pain) [7, 183]. Instead, they should promote the social interaction favoring observation of the positive effects of the therapy (e.g reduction of pain) also using video clips showing patients coping well with painful condition [50, 176, 179, 180] (Fig. 3).

Before starting the treatment clinicians should read records, thoroughly examine the patients, provide a confident diagnosis and propose, when available, different treatment options encouraging the patient’s involvement in the choice of therapy and treatment goals [169, 171, 184–186].

**Fig. 4:**
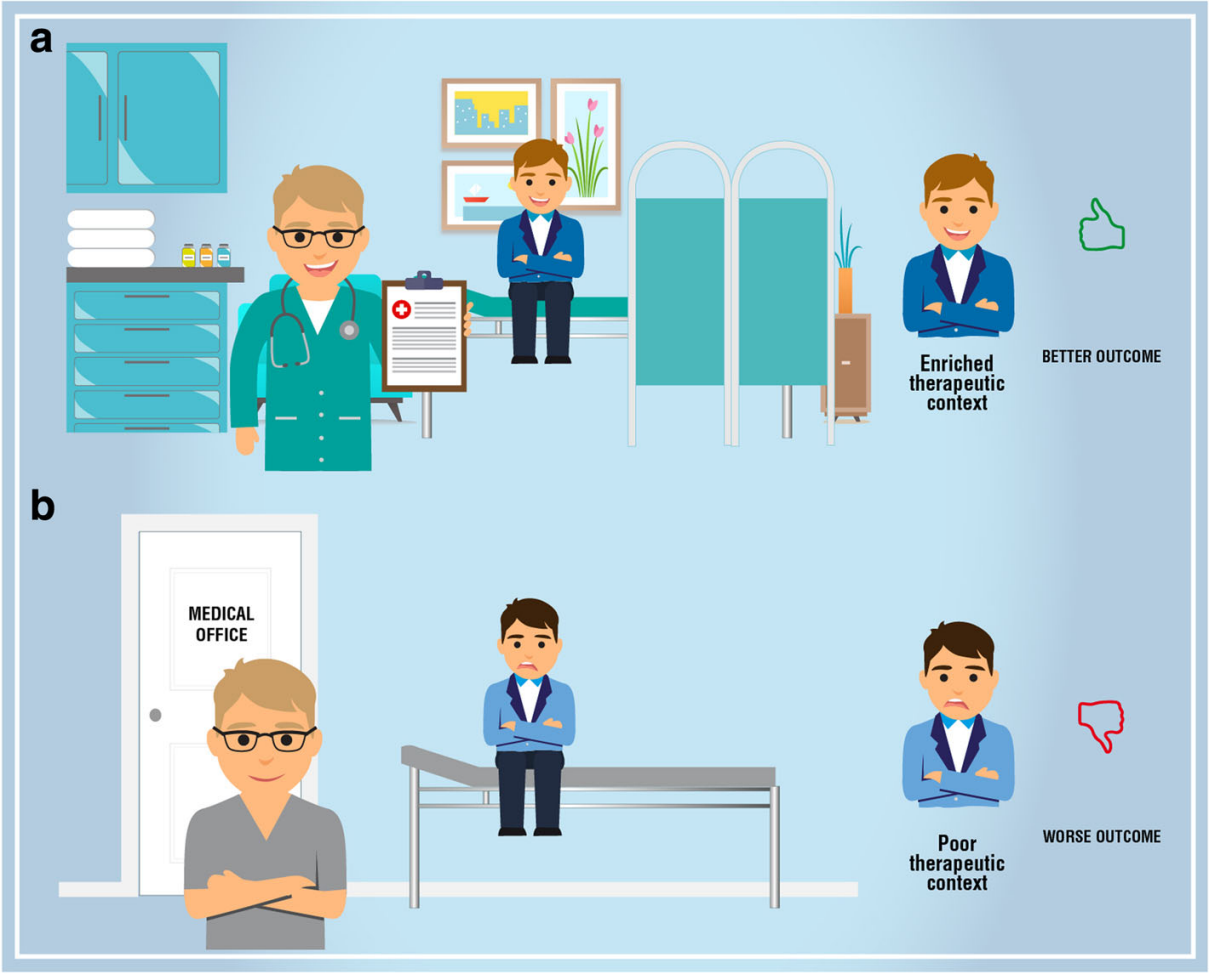
Therapeutic rituals and overt therapeutic administration. The image displays: **a** an enrich therapeutic context capable to produce positive therapeutic outcome; **b** a poor therapeutic context capable to produce negative therapeutic outcome

During treatment it is useful to avoid unintentional “hidden administration” of therapy [173]. Thus, it is crucial to focus the patient’s attention to all the salient sensory elements presented in the therapeutic arena in order to increase the contextual power of the therapy [169]. These elements are: the healthcare environment (e.g. light, color, design of the room), the physical features of the therapy (e.g. shape, size, colour, smell and taste) and the technological features of the device (e.g. novelty, price, invasiveness) [50, 169–173, 187] (Fig. 4).

After the treatment, it is valuable to assess the therapeutic outcome and give to patient a feedback on the clinical course in order to maximize the treatment adherence, encouraging the self-managing of the condition [50, 169].

The clinician’s not-verbal and verbal communication represent important element of the *overall clinical interaction* [33].

Clinicians should prepare themselves mentally and physically for the clinical encounter [38], acting as experts in their field [169, 172]. It is crucial to effectively inform about the efficacy of a specific treatment [169, 172], considering that beliefs and behaviors could influence patients’ attitudes in a positive or negative way [7, 169]. It is suitable to individualize consultation style according to the patient’s preference opting for a personal interaction and seeking for a warm, authentic and empathic style, limiting technical contacts to the minimum [169, 171, 172, 176, 188–190].

Also, the content of the message (what), the modality of delivering (how) and the time of communication (when) represent a great clinical enigma [191] and should be taken into account. It is recommended to enhance the positive expectation toward the treatment and limit the emphasis on contraindications, tell patients about side effects, but associated with positive clinical outcome. Side effects of treatment should be presented in form of probability instead of a mere list and during the informed consent process positive and negative information should be balanced [7, 50, 169, 172, 173, 176, 180, 183, 191, 192].

### What is the concern about ethics?

The adoption of placebo strategies seems to be common practice in clinical routine among healthcare providers [193]. In musculoskeletal field, orthopedics surgeon, rheumatology physicians and nurses thought that placebo effects are real, have therapeutic benefits, and are permissible within the ethical borders [194–196]. Patients with chronic musculoskeletal pain and rheumatologic complaints know what placebo effects are, consider placebo treatments acceptable when adopted as complementary/adjunct treatments and when no other established treatments are available. However, they present a lack of understanding of nocebo effects [196–198]. Scientific community is still focusing the debate on the possibility of a transparent disclosure to patients of placebo treatments [199–202]. The current researches suggest the possibility to openly prescribe sham medication or sham physical treatments with advanced prior consent [169]. Thus, when available the choice of the best evidence-based therapy is mandatory and a patient must be informed about the use of a placebo intervention with an amount of disclosure sufficient to avoid deception [201, 203, 204]. Although it is common thought that revealing the use of a placebo inhibits its effect, different studies point out the efficacy of placebo interventions also in “open label” conditions where the use of a placebo was disclosed in patients with chronic low back pain [205, 206]. From a clinical perspective, the mindful manipulation of CFs represents a useful opportunity to enrich a well-established therapy that have different ethical implication in comparison with the replacement of real treatment with a potentially ineffective treatment [200].

### Is there a place for a translational research on CFs?

There is a strong need of research studies on CFs close to routine and real-world clinical practice [49, 207] in order to underline the uncertainty of therapy action [208] and help clinicians to implement knowledge in daily practice.

The research community should investigate the effect of the different CFs on therapeutic outcome, instead of minimizing or labeling them exclusively as confounders [209, 210]. The search for a good placebo control in musculoskeletal pain field (e.g. physical therapy) represents an unresolved challenge [211, 212]. Indeed, medical treatments are generally more complex than the mere administration of a drug, involving multiple treatment components that interact with each other and that are difficult to separate (e.g. verbal instruction and education, patient-therapist contact, physical action by the patient or therapist, and sensory feedback) leading to biased estimates of treatment effect [213].

In clinical trial there is a urge to measure patient’s expectation before, during, and after the treatment [214] evaluating by standardized and validated scale all the dimensions of expectation (optimism, pain catastrophizing, hope, trust, worry and neuroticism) [215, 216]. Also measuring the impact of CFs from the patient’s perspective represents a desirable outcome to be implemented in the future researches. Recently, a new item banks (Healing Encounters and Attitudes Lists - HEAL) was proposed as suitable for measuring CFs of the treatment and present promising evidence of predictive and concurrent validity [217].

Despite CFs play a key role in pain [20, 21], there is a still paucity of knowledge on their effects in different musculoskeletal diseases, in young and old participants [218–220], in acute and chronic conditions [141], in different pain mechanism such as nociceptive, neuropathic, central sensitization [221]. It is of paramount importance to try to identify psychological, neuroendocrine or genetic elements that predict the responsiveness to specific CFs [50]. Finally, the use of meta-analysis may help to estimate the effects of the CFs [222].

### Limitations

This debate presents some limitations. The framework adopted [17, 33] for reviewing the role of the CFs was not preliminarily validated for its specific consistency in the musculoskeletal field and some factors are not related exclusively to musculoskeletal pain literature but refer to pain in general. Examples of primary studies and data offered to sustain each factors of the model were not selected by adopting a systematic review approach and not criticized in depth, given that the main goal was to propose a short synopsis. CFs have been categorized into a conceptual framework by describing each factor involved, therefore interpretations about the relationships between factors and placebo/nocebo effects need additional critical analysis and discussion.

## Conclusion

This debate points to a conscious use of the CFs, as supplementary therapeutic strategy for pain management capable to improve analgesia and prevent hyperalgesia. The good news is that pain perception can be positively influenced by an honest and aware use of CFs. The bad news is related to the complexity of the phenomenon, to a certain degree of uncertainty in the individual response and to a risk of patient’s deception associated with their use. Nevertheless, clinicians have already enough comprehensive scientific information that allows them to choose the correct behavior wisely and adjust the CFs of the therapeutic setting in an evidence-based and ethically respectful perspective. We think that time has come for clinicians to manage conscientiously and ethically the CFs to enhance the placebo and avoid nocebo effects for the benefit of their patients.

## Abbreviations

CCK: Cholecystokinin
CFs: Contextual factors
DLPFC: Dorsolateral prefrontal cortex
EEG: Electroencephalography
ES: Effect size
PAG: Periaqueductal gray
PET: Positron emission tomography
rACC: Rostral anterior cingulate cortex
RVM: Rostral ventromedial medulla
